# The clopidogrel after surgery for coronary artery disease (CASCADE) randomized controlled trial: clopidogrel and aspirin versus aspirin alone after coronary bypass surgery [NCT00228423]

**DOI:** 10.1186/1468-6708-6-15

**Published:** 2005-10-11

**Authors:** Alexander Kulik, Michel Le May, George A Wells, Thierry G Mesana, Marc Ruel

**Affiliations:** 1 Division of Cardiac Surgery, University of Ottawa Heart Institute, Ottawa, Canada; 2 Division of Cardiology, University of Ottawa Heart Institute, Ottawa, Canada; 3 Department of Epidemiology and Community Medicine, University of Ottawa, Ottawa, Canada; 4 Division of Cardiac Surgery, University of Ottawa Heart Institute, Ottawa, Canada; 5 Division of Cardiac Surgery, University of Ottawa Heart Institute, and Department of Epidemiology and Community Medicine, University of Ottawa, Ottawa, Canada

**Keywords:** coronary artery bypass graft surgery, antiplatelet therapy, saphenous vein

## Abstract

**Background:**

Saphenous vein graft disease remains a major limitation of coronary artery bypass graft surgery. The process of saphenous vein intimal hyperplasia begins just days after surgical revascularization, setting the stage for graft atherosclerotic disease and its sequalae. Clopidogrel improves outcomes in patients with atherosclerotic disease, and is effective at reducing intimal hyperplasia in animal models of thrombosis. Therefore, the goal of this study will be to evaluate the efficacy of clopidogrel and aspirin therapy versus aspirin alone in the prevention of saphenous vein graft intimal hyperplasia following coronary artery bypass surgery.

**Methods:**

Patients undergoing multi-vessel coronary artery bypass grafting and in whom at least two saphenous vein grafts will be used are eligible for the study. Patients will be randomized to receive daily clopidogrel 75 mg or placebo, in addition to daily aspirin 162 mg, for a one year duration starting on the day of surgery (as soon as postoperative bleeding has been excluded). At the end of one year, all patients will undergo coronary angiography and intravascular ultrasound assessment of one saphenous vein graft as selected by randomization. The trial will be powered to test the hypothesis that clopidogrel and aspirin will reduce vein graft intimal hyperplasia by 20% compared to aspirin alone at one year following bypass surgery.

**Discussion:**

This trial is the first prospective human study that will address the question of whether clopidogrel therapy improves outcomes and reduces saphenous vein graft intimal hyperplasia following cardiac surgery. Should the combination of clopidogrel and aspirin reduce the process of vein graft intimal hyperplasia, the results of this study will help redefine modern antiplatelet management of coronary artery bypass patients.

## Background

Coronary artery bypass graft surgery (CABG) is the most durable approach for the treatment of ischemic heart disease [1], with >400,000 procedures performed annually in the United States alone [2]. Despite the increasing application of arterial conduits during CABG, the saphenous vein remains the most common conduit, employed for more than 70% of grafts [3]. However, saphenous vein graft (SVG) disease presents an important clinical problem. Even with aggressive medical therapy [4-11], up to 15% of vein grafts occlude in the first year after bypass surgery. Between 1 and 6 years, the graft attrition rate is 1% to 2% per year, and between 6 and 10 years it rises to 4% per year. By 10 years after surgery, only 60% of grafts are patent and only 50% of patent vein grafts are free of significant stenosis. In addition, native coronary artery disease progresses in 5% of patients annually [12-15]. Reflecting the graft and native vessel attrition, this population is at high risk for subsequent ischemic events, including death, myocardial infarction (MI) and stroke [14]. Further revascularization, either reoperation or percutaneous coronary intervention, is required in 4% of patients by 5 years, 19% of patients by 10 years and 31% of patients by 12 years after the initial bypass surgery [3,16].

The process of SVG disease is composed of three mechanistically interlinked stages: thrombosis, intimal hyperplasia, and atherosclerosis [13,14,16-18]. Early graft thrombosis can occur at the time of surgery secondary to focal endothelial disruption [19,20]. Grafts that survive this early period develop a progressive thickening of the media that begins within days after implantation. This process, termed intimal hyperplasia, is a consequence of smooth muscle cell proliferation and extracellular matrix protein synthesis [21,22]. Platelets play a fundamental role in the process of smooth muscle cell proliferation and intimal hyperplasia [23,24]. Intimal hyperplasia is present in all grafts 1 month after implantation [25] and forms a template for the development of superimposed atherosclerotic changes [17,18]. With the passage of a sufficient period of time, the thrombotic occlusion of vein grafts is almost inevitable due to progressive atherosclerosis [17].

Despite its established benefit in patients with coronary artery disease, aspirin therapy has numerous limitations. It is a relatively weak antiplatelet agent and has no effect on thrombin, which is believed to play a major role in acute coronary syndrome [26]. Even with aspirin therapy for secondary prevention, a large number of recurrent events occur [27]. A significant proportion of patients undergoing CABG may be aspirin resistant, defined as undetectable platelet inhibition after one week of therapy [28,29]. Depending on the population studied and the specific definition of aspirin resistance, anywhere from 10–40% of patients appear to have an inadequate antiplatelet response to aspirin [28,30]. Such patients appear to be at increased risk for the development of vascular events. In theory, these aspirin-resistant patients may derive particular benefit from additional antiplatelet therapy [31].

Clopidogrel is a thienopyridine antiplatelet agent that inhibits ADP-dependent platelet activation and aggregation [32]. Sevenfold more potent than ticlopidine, clopidogrel is free of its adverse side effects such as neutropenia, diarrhea and rash [33]. Unlike aspirin [24,34], clopidogrel has been shown to inhibit the process of platelet-mediated intimal proliferation and smooth muscle hyperplasia in laboratory experiments. In a cell culture model, clopidogrel significantly inhibited platelet adhesion to immobilized fibrinogen and also inhibited platelet-dependent mitogenic signaling and DNA synthesis in cultured coronary artery smooth muscle cells [35]. Similarly, in animal thrombosis models, clopidogrel but not aspirin significantly inhibited platelet-mediated intimal proliferation and smooth muscle hyperplasia [33,36]. Furthermore, the combination of clopidogrel with aspirin led to potent synergistic antithrombotic effects and a decrease in myointimal proliferation compared to either therapy alone [24,36,37].

Several large clinical trials have demonstrated that clopidogrel reduces ischemic events and mortality in patients with coronary and vascular disease [38-41]. In the CAPRIE (Clopidogrel versus Aspirin in Patients with Ischemic Events) trial, clopidogrel (75 mg/day) was demonstrated to be significantly more effective than aspirin (325 mg/day) in preventing vascular thrombotic events (ischemic stroke, MI or vascular death) in patients with clinical evidence of atherosclerotic disease (clopidogrel 9.78% vs. aspirin 10.64%, relative risk reduction [RRR] 8.7%, p = 0.045) [38]. In patients presenting with acute coronary syndromes, the CURE (Clopidogrel in Unstable angina to prevent Recurrent ischemic Events) study demonstrated that the combination of clopidogrel and aspirin was more effective at reducing the primary outcome (cardiovascular death, nonfatal MI or stroke) compared to aspirin alone (clopidogrel and aspirin 9.3% vs. aspirin alone 11.4%, RRR 20%, p < 0.001) [40]. Subgroup analysis from these trials suggested that patients that underwent surgical revascularization also benefited from clopidogrel [39,42,43]. However, no trial to date has prospectively evaluated the combined effects of clopidogrel plus aspirin on saphenous vein graft disease after CABG.

There currently exists a clinical equipoise regarding the optimal antiplatelet therapy for patients who have undergone coronary artery bypass surgery. While some clinicians believe in the beneficial effects of clopidogrel, the increased risk of bleeding and the lack of data in CABG patients make it impossible to establish definitive recommendations. We therefore propose the Clopidogrel after Surgery for Coronary Artery Disease (CASCADE) study, a randomized, placebo-controlled trial comparing clopidogrel plus aspirin versus aspirin alone in CABG patients revascularized with saphenous vein. The primary aim of this study will be to evaluate the effect of combined antiplatelet therapy on the reduction of SVG intimal hyperplasia one year after CABG, through the assessment of intimal area by intravascular ultrasound (IVUS). Secondary aims will evaluate the safety of clopidogrel administration following CABG with regards to bleeding complications. We hypothesize that the combination of clopidogrel with aspirin will reduce the SVG intimal hyperplasia (intimal area) by 20% one year post-CABG compared to the usual antiplatelet therapy of aspirin alone.

## Methods

### Study Population and Recruitment Procedure

The study population will include all patients undergoing multi-vessel elective or urgent CABG using at least two saphenous vein grafts at the University of Ottawa Heart Institute (OHI) over the study period (see Table 1 for inclusion and exclusion criteria). Patients undergoing off-pump CABG (OPCAB) will also be eligible for this study, as long as at least two saphenous vein grafts are used. OPCAB is performed in order to avoid the hazards associated with standard CABG, such as cardiopulmonary bypass and aortic cross-clamping, and is carried out at the discretion of the surgeon in patients deemed to be at higher risk of thromboembolic or renal complications during surgery. All CABG patients at the OHI will be triaged pre-operatively, and study eligible patients will be selected and approached by the study nurse to explain the trial and obtain consent.

### Description of Intervention and Control

A prospective randomized double-blinded placebo-controlled study will be conducted from November 2005 to November 2007 in order to achieve the study objectives. Patients will be recruited over the first 12 months of the study, and graft evaluation for each patient will occur over the following 12 months (one year after surgery for each patient). Patients will be randomized into an experimental group (receiving clopidogrel) or a control group (placebo). The placebo and clopidogrel medications will be prepared by the Bristol Myers-squibb Sanofi Canada Partnership and appear identical. Medication administration and data collection will be performed in a double-blind manner, such that neither the patient nor the healthcare personnel will be aware of the medication assignment. Recruitment and written consent will be performed prior to surgery. However, patients will not be randomized until after surgery has been completed and clinical stability ensured. Patients that are bleeding excessively after surgery (chest tube output > 200 cc/hr) or those requiring high levels of hemodynamic support (more than 2 inotropes and/or intra-aortic balloon pump) will not be randomized into the study.

**Figure 1:**
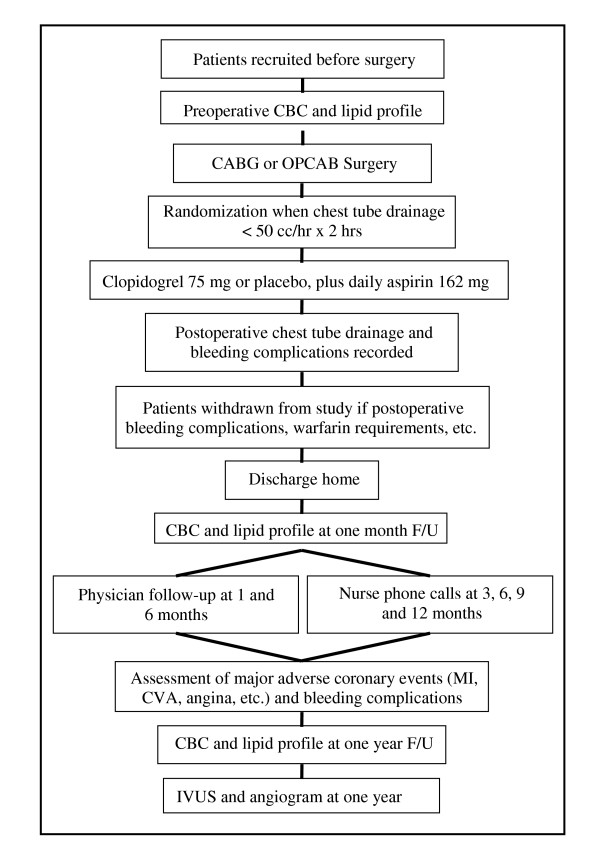
text

After surgery, the study medication will be administered via nasogastric tube when the chest tube drainage has decreased to ≤50 cc/hr for 2 hours. Each patient will receive either clopidogrel 75 mg or placebo, in addition to enteric coated aspirin 162 mg (Figure 1). The study drug and aspirin 162 mg will be repeated orally in the same dose once daily for the duration of one year. Because post-CABG patients are relatively aspirin resistant following surgery, the aspirin dose of 162 mg will ensure adequate platelet inhibition in those patients randomized to aspirin alone. Furthermore, 162 mg falls within the safety window of combining aspirin with clopidogrel [30,40,44].

### Allocation Procedure

A stratified random design allocation will be utilized to account for the presence or absence of diabetes, as well as the use or nonuse of cardiopulmonary bypass (standard CABG versus OPCAB). A block randomization technique will ensure an equal distribution of diabetic patients in both arms of the trial and an equal distribution of OPCAB patients. The randomization schedule will be generated using SAS 9.1 software (SAS, Cary, NC). All patients and study personnel will be blinded to the treatment assignment, which will be performed by the hospital pharmacy.

### Concomitant Medication and Treatments

Patients will receive concomitant therapies in both groups as recommended by the current American College of Cardiology / American Heart Association guidelines. This will include smoking cessation counseling and the administration of aspirin, beta blockers, angiotensin converting enzyme inhibitors, and lipid lowering medications. Target LDL values will be those recommended as per current guidelines [45-47] and will be assessed during the study follow-up period. Routine peptic ulcer prophylaxis will not be administered in order to fully evaluate gastrointestinal side effects.

Diabetic patients will be eligible for enrollment in this study, regardless of their preoperative need for insulin therapy, and will be allocated equally into both groups through stratified randomization. Diabetic patients will have aggressive perioperative glycemic control, including an intravenous insulin infusion both in the operating room and in the intensive care unit, and a subcutaneous insulin sliding scale while recovering on the surgical ward. Once drinking well, diabetic patients will be restarted on their original preoperative diabetic regimens (oral agents and/or insulin therapy). The treatment of diabetes during this study will be closely monitored in collaboration with an endocrinologist specializing in the management of diabetes.

### Primary Outcomes

The primary endpoint of this study will be to assess whether the addition of clopidogrel to aspirin reduces intimal hyperplasia in saphenous vein grafts 12 months after bypass surgery, as assessed by IVUS. Patients will undergo IVUS imaging 12 months post-CABG, and the average intimal area in the proximal 40 mm of one vein graft per patient will be assessed.

### Secondary Outcomes

At the time of intravascular ultrasound, coronary and graft angiography will also be performed to assess vein graft patency and areas of stenosis. Although this trial will not be sufficiently powered for such a purpose, this data will be obtained whilst gathering information pertaining to the primary outcome.

Endpoints related to safety will also be documented, both at the time of surgery as well as during the one year of study drug administration. After surgery, data will be recorded regarding chest tube blood loss, blood product transfusions, bleeding requiring tube thoracostomy or sternal re-opening, perioperative MI, and gastrointestinal complications. Complete blood counts (CBC) will assess the hemoglobin level in the immediate postoperative period and during the one year follow-up. In addition, the incidence of major adverse cardiovascular events following CABG (mortality, MI, cerebrovascular accident, hospitalization for coronary ischemia, need for coronary intervention) will be recorded.

### IVUS Procedure

Intravascular ultrasound will be used to assess the area of intimal hyperplasia present in saphenous vein grafts at 12 months after surgery. IVUS differs from angiography by providing cross-sectional images of both the vessel wall and lumen with high resolution. The process of intimal hyperplasia is easily detectable and quantifiable by IVUS, but may completely escape visualization by angiography. At one year following CABG, vein graft intimal hyperplasia is universally present.

Efforts will be made to schedule all patients at 52 ± 2 weeks from the day of randomization (Figure 1). The IVUS procedure will first start with angiography of the native coronary arteries and the coronary bypass grafts. This will allow assessment of the progression of native coronary artery atherosclerosis (both in grafted and non-grafted vessels). Patency of all bypass grafts will be scored using 1) the Fitzgibbon method [14] and 2) the TIMI classification [48]. Patency of the coronary arteries will be assessed by the TIMI method.

IVUS studies will be performed using a 40 MHz imaging catheter (Atlantis^® ^SR Pro, Boston Scientific). This catheter is a monorail system and has 6F guiding catheter compatibility. All IVUS imaging will be done with the administration of unfractionated heparin (70 units per kg, minimum 4000 units) before the introduction of the guidewire into a vein graft. Each patient will have at least two vein grafts implanted at the time of surgery. However, imaging of more than one graft is not clinically advisable for safety and practical reasons. In order to minimize bias, the cardiologist performing the IVUS procedure will be blinded to the treatment allocation, and the selection of the SVG for IVUS imaging will be randomized. For this purpose, a sequence randomization scheme (based on the number of vein grafts) will produce a random sequence of numbers for each patient, such as "2, 1 and 3". These numbers will correspond to the position of the proximal anastomosis of each SVG on the ascending aorta (by increasing number from cranial to caudal, starting from 1). The graft whose random number is produced first will be selected for intubation with the IVUS catheter, unless 1) this particular graft showed more than 50% stenosis on the selective angiography performed immediately prior, or 2) access to this graft is technically difficult (graft tortuosity, difficult graft intubation). If any of the above two instances is encountered, the graft with the next number in the randomization sequence will instead be selected for IVUS, and so on. Graft exclusion for whatever basis (either because >50% stenosis or technically difficult access) will be recorded for all patients in each group.

Once the randomized graft selection has been completed, the actual IVUS procedure will take place. Intracoronary nitroglycerin (200 μg) will be given before advancing the IVUS catheter, and a 6F guiding catheter will be used to engage the vein graft. Then, a 0.014" coronary angioplasty guidewire will be advanced distally through the vein graft and positioned into the native coronary artery. The IVUS catheter will then be advanced into the graft at least 50 mm beyond the aorto-ostial anastomosis. The guiding catheter will then be disengaged to ensure visualization of the aorto-ostial anastomosis on pullback. This is essential because the aorto-ostial anastomosis will be the only landmark available to assure measurements of the proximal portion of each graft are comparable between groups. IVUS imaging will be done using a validated motorized pullback device at 0.5 mm/sec. Each study will be recorded on a separate S-VHS videotape. Only one quality pullback will be needed per graft, but a second pullback may be repeated if the first set of pullback images were judged suboptimal in quality.

### IVUS Analysis

The IVUS images will be sent to an independent core laboratory and interpreted by an experienced cardiologist blinded to treatment allocation. The methods for the analysis have been previously reported and validated [49]. Briefly, using the aorto-ostial anastomosis as a landmark, the most proximal 40 mm of vein graft will be analyzed. The video images will be digitized and measurements of lumen, intimal hyperplasia, and external elastic lamina areas will be available for each digitized cross section. Intimal hyperplasia volumes will be computed by multiplying the corresponding areas of each of the cross-sections by the distance between slices and by adding the products. For the purpose of this study, the mean plaque area per patient for the 40 mm-analyzed segment will be used for comparison between treatment groups.

### Sample Size

The trial will be powered to test the hypothesis that clopidogrel and aspirin should reduce vein graft intimal hyperplasia by 20% compared to aspirin alone at one year following bypass surgery. According to Hozumi et al., at one year, the mean intimal area of angiographically normal saphenous vein grafts is 5.26 mm^2^, with a standard deviation of 1.38 mm^2 ^[50]. Intravascular ultrasound imaging will be performed at follow-up in one vein graft per patient. In order to account for potential angiographic refusals and study withdrawals, approximately 100 patients in total will be required to test the null hypothesis with an α value of 0.05 and a power of 0.90.

### Data Collection and Safety Monitoring

Intimal area will be recorded at the time of IVUS one year after CABG. Vein graft patency and stenosis will be assessed by angiography. The incidence of major adverse coronary events and bleeding complications will be documented during postoperative clinic visits at one month, six months and twelve months after surgery. Telephone home assessments every three months will also be used to document events. All serious adverse events will be reported to the ethics committee. The development of serious adverse events that might be attributable to clopidogrel will lead to the termination of the study drug.

### Ethics

The study will adhere to the highest research ethics standards of the OHI. This protocol follows the CONSORT guidelines [51] and was approved on July 19, 2005 by the University of Ottawa Heart Institute Human Research Ethics Board.

### Statistical Analysis

Vein graft intimal area, the primary outcome of the study, will be compared between the two randomization groups using two-sided Student's t tests. Vein graft patency will be compared using a Fisher's exact test. With respect to the secondary outcomes, continuous data will be compared between the two groups using two-sided Student's t tests, two-sample Wilcoxon rank-sum tests, or ANOVA as appropriate, and a Fisher's exact test will be used for categorical data. In order to assess possible interactions between patient characteristics (such as diabetes) and treatment outcomes, an exploratory analysis using multivariate linear regression will be performed. In the improbable event that all vein grafts are occluded and IVUS cannot be performed in a particular patient undergoing the one-year study, a value of five times the mean intimal area (decided *a priori*) will be assigned for that patient.

## Discussion

The CASCADE study is a novel randomized double-blind placebo-controlled trial that will help clarify the controversial issue of antiplatelet therapy following CABG surgery. Specifically, it will answer the questions of whether the addition of clopidogrel to aspirin is safe following cardiac surgery and whether it reduces saphenous vein graft intimal hyperplasia. Although subgroup analyses of previous trials have suggested a benefit of clopidogrel in cardiac surgery patients [39,42,43], no trial to date has specifically focused on the clinical or angiographic outcomes in patients treated with clopidogrel therapy immediately after surgical revascularization. Should the combination of clopidogrel and aspirin reduce the process of vein graft intimal hyperplasia, the CASCADE trial has the potential to redefine modern antiplatelet management of coronary artery bypass patients.

We believe that the strengths of this study are its randomized and blinded design. Patients will be randomized into two treatment groups, and graft selection for IVUS imaging will be randomized within each patient. Blinding will occur for all patients and health care providers, and the IVUS and angiogram images will be interpreted by an external blinded core laboratory. While the process of intimal hyperplasia may be reduced in the trial, the study will not be powered to demonstrate a difference in angiographic patency or freedom from coronary events following CABG. In order to demonstrate a difference in either of these outcomes, a considerably larger sample size and longer follow-up period would be required.

The results of this randomized trial will be generalizable to all patients undergoing standard CABG or OPCAB surgery with saphenous vein. Although the trial enrolment is limited to lower risk patients, it is anticipated that the results will also be applicable to higher risk patients with severe ischemic left ventricular dysfunction or those undergoing redo-CABG. High-risk patients participating in previous clopidogrel studies have been found to derive an enhanced benefit with the combined treatment of clopidogrel and aspirin [42]. Furthermore, there is no reason to anticipate differences in the pathophysiology of vein graft disease between high risk patients and those of the proposed study group.

## Conclusion

Saphenous vein graft disease continues to be a major limitation of surgical revascularization for coronary artery disease. The process of saphenous vein intimal hyperplasia begins just days after surgery, setting the stage for graft atherosclerotic disease and its sequalae. Clopidogrel has been demonstrated to improve outcomes in patients with coronary and vascular disease, and it is effective at reducing intimal hyperplasia in animal models of thrombosis. However, no prospective study to date has been conducted in humans regarding the use of clopidogrel after CABG to prevent vein graft intimal hyperplasia. The CASCADE trial is a randomized, placebo-controlled trial comparing clopidogrel plus aspirin versus aspirin alone in CABG patients revascularized with saphenous vein. The effects of clopidogrel on vein graft intimal hyperplasia will be studied with coronary angiography and intravascular ultrasound one year following CABG.

## Abbreviations

CABG – coronary artery bypass graft surgery

CASCADE – Clopidogrel after Surgery for Coronary Artery Disease Trial

CBC – complete blood counts

IVUS – intravascular ultrasound

MI – myocardial infarction

OHI – University of Ottawa Heart Institute

OPCAB – off-pump coronary artery bypass graft surgery

RRR – relative risk reduction

SVG – saphenous vein graft

## Competing interests

The author(s) declare that they have no competing interests.

## Authors' contributions

All authors read and approved the final manuscript. Specifically, AK conceived of the study, participated in the protocol design and helped draft the manuscript. MLM participated in the protocol design, helped draft the manuscript, and will perform the intravascular ultrasound procedures on study patients. GAW helped draft the manuscript, design the study protocol, and will coordinate the statistical analysis. TGM helped design the study protocol and draft the manuscript. MR is the principle investigator and helped conceive the study, design the protocol and draft the manuscript.

**Table 1:** Study Inclusion and Exclusion Criteria

***Inclusion Criteria:***	***Reason:***
- Patients undergoing primary multi-vessel CABG with at least two saphenous vein grafts, with or without cardiopulmonary bypass	- The population of clinical interest
***Preoperative Exclusion Criteria:***	***Reason:***
- Emergency surgery	- Unable to obtain consent
- Valve surgery	- Requirement of postoperative anticoagulation
- Redo CABG	- Higher risk of postoperative bleeding and low cardiac output syndrome
- Left ventricle ejection fraction <25%	- Higher risk of postoperative low cardiac output syndrome and mortality
- Serum creatinine >130 μmol/L	- Contraindication to use of postoperative angiography
- Preoperative use of clopidogrel (with the exception of the current admission)	- Contraindication to randomization, confounding of results
- Preoperative use of warfarin	- Requirement of postoperative anticoagulation
- Allergy to aspirin or clopidogrel	- Contraindication to use of aspirin or clopidogrel
- History of cerebrovascular accident	- Concurrent indication for clopidogrel
- History of severe liver disease	- Contraindication to use of clopidogrel
- Morbid obesity	- Unable to perform postoperative angiography
- Residence outside of Ottawa region	- Unable to perform postoperative angiography
- Current malignancy	- Higher risk of early postoperative mortality
- Inability to provide informed consent	- Ineligible for research study enrollment

***Postoperative Exclusion Criteria:***	***Reason:***
- Low cardiac output syndrome with inotropic support for greater than 24 hours	- Unstable patient unlikely to benefit from treatment
- Recurrent ventricular arrhythmias	- Unstable patient unlikely to benefit from treatment
- Intubation for more than 24 hours	- Unstable patient unlikely to benefit from treatment
- Postoperative bleeding or cardiac tamponade requiring surgical exploration	- Contraindication to use of clopidogrel
- Postoperative gastrointestinal bleeding	- Contraindication to use of clopidogrel
- Postoperative warfarin requirement	- Contraindication to use of clopidogrel
